# Income and gambling disorder: A longitudinal matched case-control study with registry data from Norway

**DOI:** 10.1016/j.ssmph.2023.101504

**Published:** 2023-09-08

**Authors:** Lisa-Christine Girard, Tony Leino, Mark D. Griffiths, Ståle Pallesen

**Affiliations:** a Department of Psychosocial Science, University of Bergen, Norway; b Department of Special Needs Education, Oslo University, Norway; c Department of Health Promotion, Norwegian Institute of Public Health, Norway; d Department of Psychology, Nottingham Trent University, UK; e Norwegian Competence Centre for Gambling and Gaming Research, University of Bergen, Norway

**Keywords:** Gambling disorder, Income, Norway, Case-control, Registry data, Group-based trajectory models

## Abstract

**Background:**

Untangling the association between gambling disorder (GD) and income is complex. Financial strain is often a consequence of GD. At the same time GD is more prevalent in the context of poverty, suggesting income may be a risk marker for GD.

**Aims:**

The aim of the present study was to investigate whether income is a risk marker for GD and whether the longitudinal average predicted income for patients with GD between 2008 and 2018 compared to control groups. The study also explored the potential heterogeneity in income trajectories for patients with GD and associated characteristics.

**Methods:**

A matched case-control longitudinal study was conducted using two Norwegian registries (i.e., the Norwegian Patient Registry and the Division of Welfare Statistics). A total of 65,771 participants were included, 5131 who were diagnosed with GD (cases), 30,467 diagnosed with any other psychiatric or somatic disorder (control), and 30,164 from the general population (control). Multinomial and ordinary least squares regressions, along with group-based trajectory models were estimated.

**Results:**

Individuals with GD were more likely to have income levels in the bottom quartile of the nationally reported average income in 2008 compared to the general population. However, this was not observed in the psychiatric/somatic group. Both GD and psychiatric/somatic groups were less likely to have average/above average income compared to the general population. Expected income for patients with GD was below national averages between 2008 and 2018, with significant group differences identified. Estimated trajectories for patients with GD resulted in a seven-group model. Males were more likely to have membership in higher income groups, whereas females and younger GD patients were more likely to belong to trajectory groups with the lowest income.

**Conclusion:**

The results suggest income is a risk marker for GD. Heterogeneity present across the income distribution for patients with GD, coupled with identifiable patient characteristics, may help in prediction and screening of GD.

## Highlights

• Income level across the distribution is a risk marker for gambling disorder (GD).
• Predicted income for patients with GD consistently falls below national averages over time.
• However, patients with GD evidence great heterogeneity in income trajectories.
• Male patients with GD are more likely to have higher income trajectories.
• Female and younger patients are at greatest risk for having the lowest income trajectories.

## 1 Introduction

The act of gambling involves the wagering of financial and/or other material goods of value, for which the sought outcome is of greater value, and where the real outcome is partly or completely based on chance (Potenza et al., 2001). A majority of individuals have gambled at one point or another (Potenza et al., 2001). However, for some individuals, gambling can progress into problematic and/or compulsive behaviour leading to a total loss of control and reduced quality of living. Studies have suggested a mean duration of seven to 10 years from onset of gambling behaviours prior to the transition to gambling disorder (GD) (Medeiros et al., 2017; Pettorruso et al., 2021). A clinical diagnosis of gambling disorder (GD) occurs when there are ‘frequent, repeated episodes of gambling that dominate the patient's life to the detriment of social, occupational, material, and family values and commitments’ (World Health Organization, 2016). Given that the act of gambling involves placing wagers (most often monetary), it is not surprising that finances and gambling are associated (Potenza et al., 2019).

However, untangling this association is a complex issue that remains unresolved. For instance, it is well established that financial strain and hardship are consequences of GD (e.g., due to chasing losses), often resulting in the need to seek treatment (Ledgerwood et al., 2013; Potenza et al., 2019). On the other hand, GD is more prevalent in the context of poverty, with individuals of low income experiencing higher rates of both problem gambling and GD (Calado and Griffiths, 2016; Hahmann et al., 2021). For example, estimates of problem and disordered gambling in the context of poverty range from 15% to 58% (Hahmann et al., 2021), whereas global estimates for problem gambling are reported between 0.12% and 5.8% (Calado and Griffiths, 2016). This would suggest that impoverished circumstances such as low income, may translate into risk for GD. Gambling preferences (strategic – e.g., poker, sports betting; non-strategic – e.g., slot machines, lottery, mixed) and types (online versus land-based) have also been found to be associated with income. For example, non-strategic gambling activities have been shown to be more common among individuals with higher income (Resce et al., 2019) but in contrast, strategic and mixed gambling activities have been found to occur at higher rates among individuals with lower income (Resce et al., 2019; Subramaniam et al., 2016). Online gambling has also been associated with income inequality but the direction of association has been mixed (Pabayo et al., 2023; Pallesen et al., 2021) However, while many studies have addressed associations between gambling and income levels, e.g., (Barry et al., 2007; Calado and Griffiths, 2016; Day et al., 2020; Hahmann et al., 2021; Resce et al., 2019; Subramaniam et al., 2016), there are currently few studies that have investigated income as a risk factor for GD, and none to date that have used objective registry data. This is problematic given the systematic bias in self-reported income, which may bias estimates of risk.

Previous studies have hypothesized individual level mechanisms to explain why income may be a risk factor for GD. For example, it has been suggested that gambling offers individuals with lower income the hope of upward mobility and a chance to get ahead financially (Bol et al., 2014; King, 1985); aligning with Merton's anomie theory (Merton, 1938). Additionally, heightened anxiety resulting from prolonged low-income status can alter an individual's decision-making processes (Haushofer and Fehr, 2014), resulting in higher levels of gambling as an escape or coping mechanism (Holdsworth and Tiyce, 2013). Conversely, it has been posited that higher income may also be a risk marker for developing GD given the higher levels of economic resources available (Bol et al., 2014).

### 1.1 Aims

Based on the aforementioned literature, the aims of the present study were four-fold. The first aim was to determine whether income is a risk marker for patients with GD compared to patients with any psychiatric or somatic disorder and the general population. Income has been associated with multiple psychiatric and somatic disorders (Hinz et al., 2017; Patel, 2007). Therefore, a case-control design with a group of patients diagnosed with any psychiatric or somatic disorder along with a control group from the general population affords greater context for understanding whether income as a risk factor operates in unique ways for individuals with GD. Second, the present study aimed to identify whether expected income would differ across groups longitudinally (i.e., patients with GD, any psychiatric or somatic disorder, or the general population). The third aim was to explore potential heterogeneity in the evolution of income over time for patients with GD only. The final aim was to better understand patient characteristics that may distinguish group membership in identified income trajectories.

## 2 Methods

### 2.1 Design and participants

Using a matched case-control design, participants were drawn from two registries in Norway, namely the Norwegian Patient Registry (NPR) and the Statistics of Norway (SSB), Division of Welfare Statistics. Data from these two registries were linked using participants national identity number. The NPR is a nationwide registry owned by the 10.13039/501100014232Norwegian Directorate of Health, containing information on all admissions to specialized healthcare in publicly funded hospitals/clinics in Norway (Bakken et al., 2020). Detailed health information is collected in the registry, including all disease diagnoses based on the *International Classification of Diseases, 10*th *Revision* (ICD-10), dates of all diagnoses made (month and year), the patient's national identity number, sex, and age. The SSB (Akselsen et al., 2003) collects detailed information for all residents of Norway related to their demographic and income conditions. Income conditions are gathered from tax returns and are updated annually.

All patients in the NPR with a registered diagnosis of GD (code F63.0 in the ICD-10) between 2008 and 2018 were included in the present study (*n* = 5131; males = 81.8%). The GD diagnosis was the first recorded GD diagnosis in the NPR since its establishment. Additionally, a randomly sampled control group matched on age and sex from the NPR with a registered diagnosis of any other psychiatric or somatic disorder than GD (*n* = 30,476; males = 81.6%) between 2008 and 2018 was included. A third randomly sampled control group from the SSB was also included. This group was also matched to the sample of patients with GD from the NPR on age and sex (*n* = 30,164; males = 81.4%). Ethical approval was granted by the Regional Committee for Medical and Health-Related Research Ethics in Western Norway (no. 30393).

### 2.2 Measures

The present study used annual income at the individual level which includes the sum of all income from work, property income, taxable transfers, and tax-free transfers received during the calendar year. A negative annual income is possible and occurs when an individual has realised capital losses or has had to pay back money from a transfer that was paid in the previous year but for which the individual did not have the right. Table 1 displays the annual mean income by group (i.e., GD, psychiatric/somatic, general population) between 2008 and 2018. The age of participants was categorised into three groups for analyses: 18–38 years, 39–58 years, and 59 years or older.

**Table 1:** Total annual income summary statistics between 2008 and 2018 across groups.

Gambling disorder					
	N	Mean	SD	Min	Max
Income 2008	4954	277,190.1	194,889.1	−25,000	1,025,000
Income 2009	4975	294,678.4	199,397.7	−25,000	1,025,000
Income 2010	4998	307,753.1	198,048.5	−25,000	1,025,000
Income 2011	5028	328,351.2	203,207.3	−25,000	1,025,000
Income 2012	5038	349,255.7	207,563	−25,000	1,025,000
Income 2013	5036	370,701	213,168.5	−25,000	1,025,000
Income 2014	5039	385,061.5	218,735.8	−25,000	1,025,000
Income 2015	5028	401,292.8	218,599.6	−25,000	1,025,000
Income 2016	5001	410,842.8	215,391.6	−25,000	1,025,000
Income 2017	4986	422,783.8	218,407.2	−25,000	1,025,000
Income 2018	4946	436564.9	222,534.1	−25000	1,025,000
Psychiatric/somatic					
Income 2008	22,983	314,983.9	237,530.8	−25,000	1,025,000
Income 2009	23,280	329,914.1	239,511.5	−25,000	1,025,000
Income 2010	23,621	348,301.3	244,219.4	−25,000	1,025,000
Income 2011	23,980	372,821.1	250,924.6	−25,000	1,025,000
Income 2012	24,369	396,361.2	257,421.7	−25,000	1,025,000
Income 2013	24,664	418447.9	260,800.1	−25,000	1,025,000
Income 2014	24,926	438,225.1	266,173.7	−25,000	1,025,000
Income 2015	25,060	457,553.9	266,611.2	−25,000	1,025,000
Income 2016	25,145	471,892	264,490.2	−25,000	1,025,000
Income 2017	25,214	491,147.8	263,691.7	−25,000	1,025,000
Income 2018	25,298	514,210.6	265,082.5	−25,000	1,025,000
General population					
Income 2008	23,005	324,763.1	238,860.3	−25,000	1,025,000
Income 2009	23,307	339,221	240,437	−25,000	1,025,000
Income 2010	23,600	357,786	244,821.3	−25,000	1,025,000
Income 2011	24,010	381,707.6	252,076.4	−25,000	1,025,000
Income 2012	24,400	406,053.3	257,232	−25,000	1,025,000
Income 2013	24,818	430,276.4	262,829.5	−25,000	1,025,000
Income 2014	25,190	451,004.4	265,276.7	−25,000	1,025,000
Income 2015	25,601	467,592.1	265,632.5	−25,000	1,025,000
Income 2016	25,980	479,062.7	263,518.5	−25,000	1,025,000
Income 2017	26,306	493,839.8	264,843.2	−25,000	1,025,000
Income 2018	26,367	515,814.3	267,728	−25,000	1,025,000

Note: Annual income is reported in NOK; 11 NOK is approximately equivalent to 1 USD in 2023.

### 2.3 Statistical analysis

A multinomial regression analysis was first conducted to examine whether income in 2008 was a predictor of group status (i.e., GD, psychiatric/somatic, general population), while controlling for sex and age. Income in 2008 was selected rather than income in the year of diagnosis given the mean transition time from engagement in gambling behaviour to GD. To better understand any nuanced associations, income was categorised using increments of 50,000 Norwegian Kroner (NOK) ranging between negative annual income through to more than one million NOK annually. The general population was used as the reference category. An ordinary least squares linear regression was further estimated to examine any differences in income across time for the three groups. The marginal predictions of yearly income adjusted for sex and age across groups are presented, along with Wald tests of overall and paired difference for each income year.

Next, income trajectories were estimated for the GD patient group only, allowing a better understanding of the possible heterogeneity in income evolution across time for patients with GD. Group-based trajectory modelling (GBTM), a person-centred approach using finite mixture modelling, was the preferred method because there is no assumption that trajectory parameters follow a multivariate normal distribution (Nagin, 2005). Instead, GBTM is particularly well suited for capturing heterogeneity across subgroups following distinct and potentially complex patterns of income change and/or continuity over time. A censored-normal model, with a quasi-Newton procedure, was used to estimate model parameters. Indices of model fit consisted of the Bayesian Information Criteria (BIC), the Akaike Information Criterion (AIC), the average posterior probabilities of group membership by trajectory membership group (APP), and the odds of correct classification (OCC) (of which the latter two are calculated post-model estimation). Unlike in other modelling approaches, a larger more positive value of the AIC and BIC in GBTM indicates better model fit (Nagin, 2005; Raftery, 1995). The suggested group assignment threshold for the APP and OCC are greater than 0.70 (70%) and 5, respectively (Nagin, 2005). Model selection consisted of first identifying the number of groups best fitting the data, followed by estimation of growth patterns within groups.

While the BIC and AIC favoured the eight-group model (see Table 2), closer examination identified a group with no participants. Therefore, the seven-group model was selected as best fitting the data, which was supported by the high assignment accuracy in post-model estimation using the APP and OCC indices across identified groups (see Table 3). Given the interest in participant characteristics such as sex and age, which may differentiate group membership status, a multinomial logistic regression model was also estimated to examine whether sex or age predicted group membership. All analyses were performed using Stata v17.0. The statistical threshold was set at *p* = 0.05. The term ‘significance’ is used in lieu of ‘statistical significance’ hereafter.

**Table 2:** Assessment of Model Fit: A comparison of the Bayesian Information Criterion (BIC) and the Akaike Information Criterion (AIC).

	BIC_Assessments_	BIC_Sample_	AIC
2-group	−143,117.72 (N = 55,029)	−143,110.60 (N = 5120)	−143,090.97
3-group	−135,921.16 (N = 55,029)	−135,910.48 (N = 5120)	−135,881.04
4-group	−132,721.23 (N = 55,029)	−132,706.98 (N = 5120)	−132,667.74
5-group	−130,744.56 (N = 55,029)	−130,726.75 (N = 5120)	−130,677.69
6-group	−129,622.77 (N = 55,029)	−129,601.39 (N = 5120)	−129,542.53
7-group	−129,006.90 (N = 55,029)	−128,981.96 (N = 5120)	−128,913.29
8-group	−128,445.14 (N = 55,029)	−128,416.65 (N = 5120)	−128,338.16

Note: BIC_sample_ represents the actual sample size of participants included in the trajectories, and the larger BIC_assesssments_ represents the total number of assessments used within the estimation of the model across time and participants. The two presented BIC scores bracket the theoretically correct BIC score (Nagin, 2005).

**Table 3:** Model fit criterion of income trajectories.

Trajectory Group	*n*	Average Posterior Probability of Group Membership	Odds of Correct Classification
1	868	94.3	81.1
2	605	93.2	102.9
3	1352	89.5	23.9
4	1027	89.4	33.7
5	934	86.6	29.0
6	197	93.4	354.8
7	148	97.8	1516.5

Note: Average Posterior Probability of Group Membership (i.e., the probability that a specific participant belongs to the model's *J* trajectory group) greater than 70 and an Odds of Correct Classification (i.e., the model classifies a specific participant X times better in the specified *J* trajectory group than by chance alone) greater than 5 represents good model fit. Group 1 refers to lowest income earners, Group 2 refers to average increasing to high income earners, Group 3 refers to low stable earners, Group 4 refers to average income earners (reference group), Group 5 refers to low increasing to average earners, Group 6 refers to stable high earners, Group 7 refers to highest income earners.

## 3 Results

A test of the full multinomial regression model against the constant only model was significant, indicative of an improved model fit in predicting group membership, *χ*^2^ (48) = 537.60, *p* < 0.001. Results suggested that as compared to the general population, patients with GD were significantly more likely to have an annual income bracket between 100,000 NOK and up to 350,000 NOK with the relative risk ratios (RRRs), ranging between 1.31 and 1.72, while significantly less likely to have an annual income bracket over 450,000 NOK with RRRs ranging between 0.76 and 0.39. In contrast, patients with any psychiatric or somatic disorder were generally less likely to have an annual income bracket between 350,000 and 700,000 NOK with RRRs ranging between 0.92 and 0.78. However, no significant differences were observed for income in the lowest and highest brackets when compared to the general population for the psychiatric/somatic group. See Table 4 for full model results.

**Table 4:** Multinomial logistic regression: Income as a predictor of group status.

	RRR	S.E.	*p*-value	Lower 95 CI	Upper 95 CI
**Gambling disordersample vs. General population(reference group)**					
Negative income	1.87	0.61	0.057	0.98	3.56
50,001–100,000	1.09	0.08	0.228	0.95	1.27
100,001–150,000	1.31	0.10	>0.001	1.14	1.52
150,001–200,000	1.59	0.11	>0.001	1.39	1.82
200,001–250,000	1.72	0.12	>0.001	1.50	1.97
250,001–300,000	1.68	0.11	>0.001	1.48	1.92
300,001–350,000	1.39	0.09	>0.001	1.22	1.58
350,001–400,000	1.02	0.07	0.763	0.89	1.17
400,001–450,000	0.96	0.07	0.569	0.83	1.11
450,001–500,000	0.76	0.07	0.002	0.64	0.90
500,001–550,000	0.75	0.08	0.005	0.62	0.92
550,001–600,000	0.67	0.08	0.001	0.53	0.85
600,001–650,000	0.62	0.08	>0.001	0.48	0.81
650,001–700,000	0.49	0.08	>0.001	0.35	0.69
700,001–750,000	0.48	0.09	>0.001	0.33	0.70
750,001–800,000	0.39	0.09	>0.001	0.25	0.62
800,001–850,000	0.48	0.12	0.004	0.29	0.79
850,001–900,000	0.46	0.13	0.005	0.27	0.79
900,001–950,000	0.54	0.16	0.040	0.30	0.97
950,001–1,000,000	0.39	0.16	0.018	0.18	0.85
More than 1 million	0.44	0.07	>0.001	0.32	0.59
Male	1.30	0.05	>0.001	1.19	1.41
18–38 years	0.96	0.04	0.357	0.89	1.04
59 years or more	0.88	0.05	0.029	0.79	0.99
Constant	0.17	0.01	>0.001	0.15	0.19
**Psychiatric/somatic sample vs. General population(reference group)**					
Negative income	1.39	0.31	0.134	0.90	2.14
50,001–100,000	0.99	0.04	0.804	0.91	1.08
100,001–150,000	1.03	0.05	0.487	0.94	1.13
150,001–200,000	0.98	0.04	0.633	0.90	1.07
200,001–250,000	0.97	0.04	0.482	0.89	1.06
250,001–300,000	0.95	0.04	0.210	0.87	1.03
300,001–350,000	0.95	0.04	0.171	0.87	1.03
350,001–400,000	0.92	0.04	0.042	0.85	0.99
400,001–450,000	0.90	0.04	0.011	0.82	0.98
450,001–500,000	0.87	0.04	0.004	0.80	0.96
500,001–550,000	0.89	0.05	0.034	0.80	0.99
550,001–600,000	0.91	0.06	0.129	0.81	1.03
600,001–650,000	0.82	0.06	0.003	0.72	0.94
650,001–700,000	0.78	0.06	0.001	0.67	0.91
700,001–750,000	0.91	0.08	0.291	0.77	1.08
750,001–800,000	0.90	0.09	0.281	0.75	1.09
800,001–850,000	0.95	0.10	0.655	0.77	1.18
850,001–900,000	0.88	0.10	0.258	0.69	1.10
900,001–950,000	1.04	0.14	0.760	0.81	1.35
950,001–1,000,000	0.76	0.12	0.084	0.55	1.04
More than 1 million	0.85	0.06	0.021	0.74	0.98
Males	1.10	0.03	>0.001	1.05	1.15
18–38 years	0.97	0.02	0.151	0.92	1.01
59 years or more	0.95	0.03	0.147	0.90	1.02
Constant	1.00	0.04	0.926	0.93	1.08

Note: Annual income is reported in NOK; 11 NOK is approximately equivalent to 1 USD in 2023. RRR refers to relative risk ratio. The reference category for age is 39–58 years of age. The reference category for annual income is 0–50,000 NOK.

**Fig. 1:**
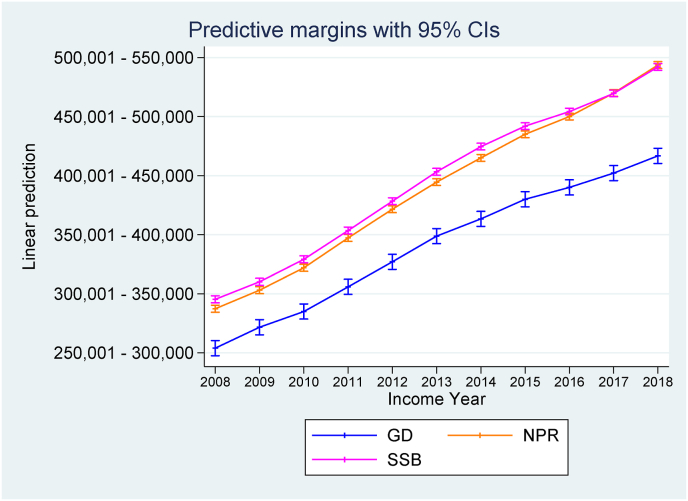
Predictive margins for average income across groups.

The predictive margins and 95% confidence intervals (CIs) of annual income across groups are presented in Fig. 1. Results showed an average increase in income for all groups between 2008 and 2018. However, patients with GD (on average) started at a lower predicted annual income compared to the patients with any psychiatric or somatic disorder and the general population. Wald tests of overall difference showed that prior to 2013, the differences in income across all three groups was not statistically significant. Between 2013 and 2018, income level significantly differed across all groups. Paired group difference tests (i.e., the GD group and the psychiatric/somatic group, along with the GD group and the general population group) showed significant group differences from 2012 to 2018 but not before (see Table 5).

**Table 5:** Wald tests of differences in annual income across groups.

	F	df	*p*
**2009:**			
GD & Psy/som group	0.16	(1,596,118)	0.691
GD & GP	0.32	(1,596,118)	0.573
All groups	0.17	(2,596,118)	0.847
**2010:**			
GD & Psy/som group	0.52	(1,596,118)	0.470
GD & GP	0.32	(1,596,118)	0.573
All groups	0.26	(2,596,118)	0.769
**2011:**			
GD & Psy/som group	2.48	(1,596,118)	0.115
GD & GP	1.54	(1,596,118)	0.213
All groups	1.24	(2,596,118)	0.288
**2012:**			
GD & Psy/som group	4.83	(1,596,118)	0.027
GD & GP	3.86	(1,596,118)	0.049
All groups	2.45	(2,596,118)	0.086
**2013:**			
GD & Psy/som group	5.94	(1,596,118)	0.014
GD & GP	6.71	(1,596,118)	0.009
All groups	3.49	(2,596,118)	0.030
**2014:**			
GD & Psy/som group	12.95	(1,596,118)	<0.001
GD & GP	15.29	(1,596,118)	<0.001
All groups	7.85	(2,596,118)	<0.001
**2015:**			
GD & Psy/som group	18.10	(1,596,118)	<0.001
GD & GP	16.40	(1,596,118)	<0.001
All groups	9.49	(2,596,118)	<0.001
**2016:**			
GD & Psy/som group	27.26	(1,596,118)	<0.001
GD & GP	20.17	(1,596,118)	<0.001
All groups	13.67	(2,596,118)	<0.001
**2017:**			
GD & Psy/som group	45.54	(1,596,118)	<0.001
GD & GP	26.29	(1,596,118)	<0.001
All groups	23.13	(2,596,118)	<0.001
**2018:**			
GD & Psy/som group	72.76	(1,596,118)	<0.001
GD & GP	44.52	(1,596,118)	<0.001
All groups	36.66	(2,596,118)	<0.001

Note: Psy/som refers to the psychiatric/somatic group. GP refers to the general population control group.

**Fig. 2:**
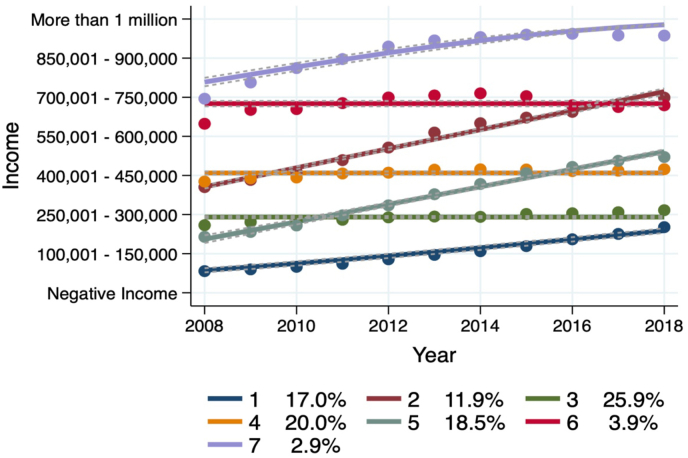
Income trajectories for patients with GD.

Seven groups following distinct income trajectories were identified among patients with a registered GD diagnosis. Group 1 (labelled ‘lowest income earners’) comprised an estimated 17.0% of the sample who, while increasing, evidenced the lowest levels of annual income across the 11-year period. Group 2 (labelled ‘average increasing to high income earners’) followed an increasing trajectory. They comprised an estimated 11.9% of the sample and while starting with an average annual income, increased steadily to high income levels. Group 3, the largest group (labelled ‘low stable earners’) comprised an estimated 25.9% of the sample. Annual income remained stably low in this group. Group 4 (labelled ‘average income earners’) comprised an estimated 20.0% of the sample. This group's income remained stable over time and was on average most similar to the reported annual average yearly earnings in Norway. Group 5 (labelled ‘low increasing to average earners’) comprised an estimated 18.5% of the sample. While annual income was initially very low (below average levels), income increased substantially to nearly the annual average by 2018. Group 6 (labelled ‘stable high earners’) comprised an estimated 3.9% of the sample. This group had stably elevated levels of annual income. Finally, Group 7 (labelled ‘highest income earners’) comprised 2.9% of the sample and followed a high increasing trajectory of income. See Fig. 2 for estimated income trajectories and Table 6 for parameter estimates of trajectory groups.

**Table 6:** Trajectory parameter estimates for the seven-group model.

Group	Parameter	Estimate	S.E.	*T*	*p*
1	Intercept	−681.83	16.46	−41.42	<0.001
	Linear	0.34	0.01	41.61	<0.001
2	Intercept	−1461.94	29.57	−49.44	<0.001
	Linear	0.73	0.01	49.79	<0.001
3	Intercept	5.80	0.03	168.07	<0.001
4	Intercept	9.19	0.06	143.25	<0.001
5	Intercept	−1349.88	32.28	−41.81	<0.001
	Linear	0.67	0.02	42.08	<0.001
6	Intercept	14.51	0.10	141.21	<0.001
7	Intercept	−1183.68	41.64	−28.43	<0.001
	Linear	0.60	0.02	28.88	<0.001
	Sigma	2.24	0.007	320.104	<0.001

Note: 5120 patients with GD were included in the trajectory model. Group 1 refers to lowest income earners, Group 2 refers to average increasing to high income earners, Group 3 refers to low stable earners, Group 4 refers to average income earners (reference group), Group 5 refers to low increasing to average earners, Group 6 refers to stable high earners, Group 7 refers to highest income earners.

To test whether participants' characteristics such as sex and age predicted group membership, a multinomial logistic regression was conducted using Group 4 (‘average income earners’), as the reference group. Full results are presented in Table 7.

**Table 7:** Sex and age as antecedents for trajectory group membership.

Trajectory Group	RRR	S.E.	*p*-value	Upper 95 CI	Lower 95 CI
**Group 1**					
Males	1.10	0.16	0.507	0.83	1.48
18–38 years	23.41	3.28	<0.001	17.78	30.82
59 years or more	2.11	0.53	0.003	1.29	3.44
**Group 2**					
Males	1.94	0.32	<0.001	1.41	2.67
18–38 years	1.63	0.18	<0.001	1.31	2.04
59 years or more	0.95	0.18	0.776	0.65	1.37
**Group 3**					
Males	0.43	0.04	<0.001	0.36	0.53
18–38 years	2.06	0.19	<0.001	1.71	2.48
59 years or more	1.56	0.21	0.001	1.19	2.04
**Group 5**					
Males	0.80	0.10	0.070	0.62	1.01
18–38 years	5.60	0.58	<0.001	4.57	6.85
59 years or more	0.73	0.16	0.138	0.48	1.10
**Group 6**					
Males	2.83	0.80	<0.001	1.63	4.91
18–38 years	0.52	0.11	0.001	0.35	0.77
59 years or more	0.78	0.22	0.365	0.45	1.34
**Group 7**					
Males	9.14	4.71	<0.001	3.34	25.07
18–38 years	0.48	0.12	0.003	0.30	0.80
59 years or more	1.89	0.46	0.009	1.17	3.05

Note: RRR refers to the relative risk ratio. The reference category for sex is females. The reference category for age is 39–58 years of age. Group 1 refers to lowest income earners, Group 2 refers to average increasing to high income earners, Group 3 refers to low stable earners, Group 4 refers to average income earners (reference group), Group 5 refers to low increasing to average earners, Group 6 refers to stable high earners, Group 7 refers to highest income earners.

## 4 Discussion

The aims of the present study were four-fold and included a better understanding of (i) income as a risk marker of GD, (ii) annual differences in income over time for patients with GD as compared to control groups, (iii) heterogeneity in income trajectories for patients with GD, and (iv) factors associated with trajectory group membership. Given the in-depth exploration of income as related to GD using objective registry data, the findings both support and add new insights to the extant literature as described below.

First, the results suggest that low income is a risk marker for GD. More specifically, patients with GD were more likely to have a lower annual income in 2008 as compared to the general population (but which was not observed between the psychiatric/somatic group and the general population). On the other hand, patients with GD were less likely to have a higher annual income (i.e., over 450,000 NOK) compared to the general population, and which was similarly observed in the psychiatric/somatic group, albeit at a lower income threshold in the latter group (i.e., 350,000 NOK). Together, this suggests that lower annual income is a risk marker specific to GD and aligns with previous empirical findings evidencing higher estimates of gambling problems and GD in the context of poverty and lower income (Hahmann et al., 2021). As has been suggested, having a lower income may create a higher risk for gambling problems and GD to develop given the potential hope that gambling offers as a means to get ahead financially (King, 1985). It is also possible that heightened anxiety related to low income status alters decision-making processes (Haushofer and Fehr, 2014) and increases engagement in gambling activities as a coping mechanism (Bol et al., 2014; Holdsworth and Tiyce, 2013).

Second, the results showed marked differences between patients with GD and the two control groups with respect to the average expected annual income across the 11-year period of investigation. More specifically, patients with GD evidenced a consistently lower average income, which notably, also fell far below the annual reported average income for all residents of Norway aged 17 years and above (e.g., 345,300 NOK in 2008, 550,800 NOK in 2018) (Holdsworth and Tiyce, 2013; Patel, 2007). Conversely, the average expected annual income for both the general population group and psychiatric/somatic group more closely mirrored the income levels reported by Statistics Norway between 2008 and 2018, albeit still falling slightly below this national average. In context, the result suggest that lower income is more prominent for patients with GD as compared to both control groups, including patients with any other psychiatric or somatic disorder. This provides important insights given that low income is a well-documented and robust risk marker for psychiatric and somatic disorders (Björkenstam et al., 2017; Kinge et al., 2021; Patel, 2007).

Third, the findings suggest a large degree of heterogeneity in income trajectories for patients with GD (i.e., seven distinct income trajectories were identified) at both ends of the income distribution. However, the majority of the sample were at the lower end of this distribution. For example, 61.4% of patients with GD followed income trajectories (i.e., Groups 1, 3, and 5) below the nationally reported average income over time, of which 42.9% were within the lowest quartile of the reported average income distribution in Norway between 2008 and 2018 (Statistics Norway, 2023a; Statistics Norway, 2023b). Conversely, only 6.8% (i.e., Groups 6 and 7) followed income trajectories categorised within the upper quartile of the reported average income distribution in Norway during this same period (Statistics Norway, 2023a; Statistics Norway, 2023b). It has been suggested that gambling provides a risk-taking outlet for leisure, which may increase the risk of gambling problems and GD for individuals in higher income groups (Bol et al., 2014), helping to explain these high income trajectory groups found in the present study.

However, given the very small percentage of the total GD cases with high income, these findings suggest that only a very marginal proportion of gamblers develop GD due to having high levels of economic resources available for gambling purposes (Bol et al., 2014). Of the seven groups identified, four followed an increasing trajectory (Groups 1, 2, 5, and 7), while three groups (Groups 3, 4, and 6) had stable income trajectories. It is noteworthy that Group 3 comprised the largest estimated group of the sample with a stable income trajectory remaining within the lowest income quartile in Norway. In the context of the inflation observed across the decade plus long period that was investigated (i.e., an average increase of 205,500 NOK), stable trajectories may actually reflect decreasing income trajectories.

Fourth, from a clinical perspective, it is important to be able to understand whether participant characteristics help explain trajectory group membership across the income distribution. The results suggest that males as compared to females had a greater risk of belonging to the three highest income trajectory groups (i.e., Groups 2, 6, and 7), with nine times greater risk for belonging to the very highest income group. On the other hand, being female increased the risk of belonging to one of the lowest income trajectory groups (i.e., Group 3), which furthermore was among one of the stable trajectories. Given that GD is more prevalent among males, this finding suggests that females with GD are a particularly vulnerable and disadvantaged group with respect to sustained lower income and financial hardship.

Age was also an important predictor of trajectory group membership. More specifically, younger age increased the risk of membership across the lowest income groups (i.e., Groups 1, 3, and 5), with the strongest probability for membership in the very lowest income trajectory (i.e., and relative risk ratio of 23.40). Conversely, younger patients with GD were less likely to belong to the two highest income trajectory groups, suggesting a very clear pattern between younger age and the spectrum of high income and low income trajectories. Older age showed less distinctive patterns. In particular, the results suggested that patients 59 years or older had an increased probability of membership in both the highest and lowest two income trajectories (i.e., Groups 1, 3, and 7).

### 5 Strengths and limitations

Some notable strengths of the study include the use of objective population-based registry data that is free from recall, non-response, and other self-reporting biases which are methodological issues of particular importance when examining questions related to clinical and economic outcomes. Moreover, the sample size was large and spanned more than a decade-long period. Finally, given that the Norwegian specialized treatment services are virtually free of charge, there is a low likelihood of systematic selection into treatment resulting from economic barriers. However, there were also some limitations of the study that must be considered when interpreting the findings. First, only treatment-seeking patients were included in the registry. While patients in the registry met specific criteria for a clinical diagnosis of GD, representativeness across the spectrum of gambling problems may be of concern. Second, and relatedly, generalisability outside treatment-seeking individuals is cautioned given differences between patients who seek treatment and individuals who do not. Third, no information was available to discern between the types or preferences of gambling activities (e.g., land-based, online gambling, strategic versus non-strategic) and income trajectories among patients with GD. This should therefore be a topic for future registry-based research. A fourth limitation relates to GD being diagnosed throughout the study period. The timing of diagnosis and treatment likely have implications for income over time. For example, prior to treatment, it might be expected that income levels would be at their lowest, whereas following treatment, if successful, income levels may gradually start to rise. Fifth, while the psychiatric/somatic control group did not have a diagnosis of GD, it was possible for patients with GD to also have a diagnosis of any psychiatric or somatic disorder. This potential comorbidity in patients with GD should be considered when interpreting these findings. Sixth, the data came only from Norway and there may be cultural differences that contribute to income divergence with other countries. For example, income differences in Norway are flatter as compared to other European and non-European countries. Finally, only information on the patient's sex and age were available to the authors. Other important factors not included in the model may have helped in further understanding characteristics that differentiate between trajectory group membership. For example, potential individual characteristics such as marital or immigration status, in addition to area-level characteristics including socioeconomic deprivation, rural-urban dwelling, and accessibility of gambling opportunities have been associated with GD (Allami et al., 2021; Hahmann et al., 2021; Syvertsen et al., 2023) and may further help to differentiate group membership, particularly related to the highest versus lowest income groups. The prevalence of GD may also vary in terms of geographical locations. However, this is currently not known in Norway.

## 6 Conclusion

Taken together, the results of the present study suggest that income is a risk marker specific to GD, evidencing distinct patterns of association for patients with GD as compared to patients with other psychiatric or somatic disorders. The heterogeneity observed across the income distribution for patients with GD, coupled with identifiable patient characteristics for income group trajectories, may help in both the prediction and screening of GD.

## Financial disclosure statement

The present study was funded by the 10.13039/501100005416Research Council of Norway, grant no. 273718. The authors have no other financial relationships relevant to this article to disclose.

## Conflicts of interest

The authors (except MDG) have no conflicts of interest relevant to this article to disclose. MDG has received research funding from *Norsk Tipping* (the gambling operator owned by the Norwegian government). MDG has also received funding for a number of research projects in the area of gambling education for young people, social responsibility in gambling and gambling treatment from *Gamble Aware* (formerly the *Responsibility in Gambling Trust*), a charitable body which funds its research program based on donations from the gambling industry. MDG undertakes consultancy for various gambling companies in the area of player protection and social responsibility in gambling.

## Credit author statement

**LCG:** Conceptualization, methodology, formal analysis, visualization, writing - original draft, review, and editing **TL:** Data curation, writing – review and editing **MDG:** Writing – review and editing **SP:** Conceptualization, methodology, writing – review and editing, supervision, project administration, funding acquisition.

## Declaration of competing interest

The authors declare that they have no known competing financial interests or personal relationships that could have appeared to influence the work reported in this paper.

## Data Availability

The authors do not have permission to share these data.

## References

[bib1] Akselsen A., Lien S., Sandnes T. (2003).

[bib2] Allami Y., Hodgins D.C., Young M., Brunelle N., Currie S., Dufour M., Nadeau L. (2021). A meta-analysis of problem gambling risk factors in the general adult population. Addiction.

[bib3] Bakken I.J., Ariansen A.M.S., Knudsen G.P., Johansen K.I., Vollset S.E. (2020). The Norwegian patient registry and the Norwegian registry for primary health care: Research potential of two nationwide health-care registries. Scandinavian Journal of Public Health.

[bib4] Barry D.T., Maciejewski P.K., Desai R.A., Potenza M.N. (2007). Income differences and recreational gambling. Journal of Addiction Medicine.

[bib5] Björkenstam E., Cheng S., Burström B., Pebley A.R., Björkenstam C., Kosidou K. (2017). Association between income trajectories in childhood and psychiatric disorder: A Swedish population-based study. Journal of Epidemiology & Community Health.

[bib6] Bol T., Lancee B., Steijn S. (2014). Income inequality and gambling: A panel study in the United States (1980–1997). Sociological Spectrum.

[bib7] Calado F., Griffiths M.D. (2016). Problem gambling worldwide: An update and systematic review of empirical research (2000–2015). J Behav Addict.

[bib8] Day B., Rosenthal G., Adetunji F., Monaghan A., Scheele C., Tracy J.K. (2020). Evaluating for differences by race/ethnicity in the association between income and gambling disorder. Journal of Gambling Studies.

[bib9] Hahmann T., Hamilton-Wright S., Ziegler C., Matheson F.I. (2021). Problem gambling within the context of poverty: A scoping review. International Gambling Studies.

[bib10] Haushofer J., Fehr E. (2014). On the psychology of poverty | Science. Science.

[bib11] Hinz A., Ernst J., Glaesmer H., Brähler E., Rauscher F.G., Petrowski K., Kocalevent R.D. (2017). Frequency of somatic symptoms in the general population: Normative values for the Patient Health Questionnaire-15 (PHQ-15). Journal of Psychosomatic Research.

[bib12] Holdsworth L., Tiyce M. (2013). Untangling the complex needs of people experiencing gambling problems and homelessness. International Journal of Mental Health and Addiction.

[bib13] King K.M. (1985). Gambling: Three forms and three explanations. Sociological Focus.

[bib14] Kinge J.M., Øverland S., Flatø M., Dieleman J., Røgeberg O., Magnus M.C., Torvik F.A. (2021). Parental income and mental disorders in children and adolescents: Prospective register-based study. International Journal of Epidemiology.

[bib15] Ledgerwood D.M., Arfken C.L., Wiedemann A., Bates K.E., Holmes D., Jones L. (2013). Who goes to treatment? Predictors of treatment initiation among gambling help-line callers. American Journal on Addictions.

[bib16] Medeiros G.C., Redden S.A., Chamberlain S.R., Grant J.E. (2017). Gambling disorder: Association between duration of illness, clinical, and neurocognitive variables. J Behav Addict.

[bib17] Merton R.K. (1938). Social structure and anomie. American Sociological Review.

[bib18] Nagin D.S. (2005).

[bib19] Pabayo R., Patel P., Patte K.A., Leatherdale S.T. (2023). Income inequality and the odds of online gambling among a large sample of adolescents in Canada. Journal of Gambling Studies.

[bib20] Pallesen S., Mentzoni R.A., Morken A.M., Engebø J., Kaur P., Erevik E.K. (2021). Changes over time and predictors of online gambling in three Norwegian population studies 2013–2019. Frontiers in Psychiatry.

[bib21] Patel V. (2007). Mental health in low- and middle-income countries. British Medical Bulletin.

[bib22] Pettorruso M., Testa G., Granero R., Martinotti G., d’Andrea G., di Giannantonio M., Baenas I. (2021). The transition time to gambling disorder: The roles that age, gambling preference and personality traits play. Addictive Behaviors.

[bib23] Potenza M.N., Balodis I.M., Derevensky J., Grant J.E., Petry N.M., Verdejo-Garcia A., Yip S.W. (2019). Gambling disorder. Nature Reviews Disease Primers.

[bib24] Potenza M.N., Kosten T.R., Rounsaville B.J. (2001). Pathological gambling. JAMA.

[bib25] Raftery A.E. (1995). Bayesian model selection in social research. Sociological Methodology.

[bib26] Resce G., Lagravinese R., Benedetti E., Molinaro S. (2019). Income-related inequality in gambling: Evidence from Italy. Review of Economics of the Household.

[bib27] Statistics Norway (2023). 08053: Mean, median and quartile monthly earnings for full-time employees, by industry section (SIC2007) and sex (closed series) 2008 - 2015. Statbank Norway. SSB. https://www.ssb.no/en/system/.

[bib28] Statistics Norway (2023). 11417: Annual earnings, by industry (SIC2007), contents and year. Statbank Norway. SSB. https://www.ssb.no/en/system/.

[bib29] Subramaniam M., Abdin E., Vaingankar J.A., Shahwan S., Picco L., Chong S.A. (2016). Strategic versus nonstrategic gambling: Results from a community survey. Journal of Addiction Medicine.

[bib30] Syvertsen A., Leino T., Pallesen S., Smith O.R., Sivertsen B., Griffiths M.D., Mentzoni R.A. (2023). Marital status and gambling disorder: A longitudinal study based on national registry data. BMC Psychiatry.

[bib31] World Health Organization (2016). https://icd.who.int/browse10/2016/en#/F63.0.

